# Challenges of Pituitary Apoplexy in Pregnancy

**DOI:** 10.3390/jcm12103416

**Published:** 2023-05-11

**Authors:** Ana-Maria Gheorghe, Alexandra-Ioana Trandafir, Mihaela Stanciu, Florina Ligia Popa, Claudiu Nistor, Mara Carsote

**Affiliations:** 1 Department of Endocrinology, “C.I. Parhon” National Institute of Endocrinology, 011683 Bucharest, Romania; anamaria.gheorghe96@yahoo.com; 2 Department of Endocrinology, Doctoral School of “Carol Davila” University of Medicine and Pharmacy, “C.I. Parhon” National Institute of Endocrinology, 011683 Bucharest, Romania; alexandratrandafir26@gmail.com; 3 Department of Endocrinology, Faculty of Medicine, “Lucian Blaga” University of Sibiu, 50169 Sibiu, Romania; 4 Department of Physical Medicine and Rehabilitation, Faculty of Medicine, “Lucian Blaga” University of Sibiu, 550169 Sibiu, Romania; florina.popa@yahoo.com; 5 Department 4—Cardio-Thoracic Pathology, Thoracic Surgery II Discipline, Faculty of Medicine, “Carol Davila” University of Medicine and Pharmacy & Thoracic Surgery Department, “Dr. Carol Davila” Central Emergency University Military Hospital, 013058 Bucharest, Romania; ncd58@yahoo.com; 6 Department of Endocrinology, “Carol Davila” University of Medicine and Pharmacy & “C.I. Parhon” National Institute of Endocrinology, 011683 Bucharest, Romania

**Keywords:** pregnancy, pituitary apoplexy, postpartum, hormone, endocrine, neurosurgery, surgery, pituitary neuroendocrine tumor, pituitary adenoma

## Abstract

Our purpose is to provide new insights concerning the challenges of pituitary apoplexy in pregnancy (PAP) and the postpartum period (PAPP). This is a narrative review of the English literature using a PubMed search. The inclusion criteria were clinically relevant original studies (January 2012–December 2022). Overall, we included 35 original studies: 7 observational studies (selected cases on PA) and 28 case reports, including 4 case series (N = 49; PAP/PAPP = 43/6). The characteristics of PAP patients (N = 43) are as follows: maternal age between 21 and 41 (mean of 27.76) years; 21/43 subjects with a presentation during the third trimester (only one case during first trimester); average weak of gestation of 26.38; most females were prim gravidae; 19 (out of 30 patients with available data on delivery) underwent a cesarean section. Headache remains the main clinical feature and is potentially associated with a heterogeneous panel (including visual anomalies, nausea, vomiting, cranial nerve palsies, diabetes insipidus, photophobia, and neck stiffness). Pre-pregnancy medication included dopamine agonists (15/43) and terguride (1/43) in addition to subsequent insulin therapy for gestational diabetes (N = 2) and type 1 diabetes mellitus (N = 1). Overall, 29/43 females received the conservative approach, and 22/43 women had trans-sphenoidal surgery (TSS) (and 10/22 had the initial approach). Furthermore, 18/43 patients had a pituitary adenoma undiagnosed before pregnancy. Most PA-associated tumors were prolactinomas (N = 26/43), with the majority of them (N = 16/26) being larger than 1 cm. A maternal–fetal deadly outcome is reported in a single case. The characteristics of PAPP patients (N = 6) are as follows: mean age at diagnosis of 33 years; 3/6 subjects had PA during their second pregnancy; the timing of PA varied between 5 min and 12 days after delivery; headache was the main clinical element; 5/6 had no underlying pituitary adenoma; 5/6 patients were managed conservatively and 1/6 underwent TSS; pituitary function recovered (N = 3) or led to persistent hypopituitarism (N = 3). In conclusion, PAP represents a rare, life-threatening condition. Headache is the most frequent presentation, and its prompt distinction from other conditions associated with headache, such as preeclampsia and meningitis, is essential. The index of suspicion should be high, especially in patients with additional risk factors such as pre-gestation treatment with dopamine agonists, diabetes mellitus, anticoagulation therapy, or large pituitary tumors. The management is conservative in most cases, and it mainly includes corticosteroid substitution and dopamine agonists. The most frequent surgical indication is neuro-ophthalmological deterioration, although the actual risk of pituitary surgery during pregnancy remains unknown. PAPP is exceptionally reported. To our knowledge, this sample–case series study is the largest of its kind that is meant to increase the awareness to the benefit of the maternal–fetal outcomes from multidisciplinary insights.

## 1. Introduction

Pituitary apoplexy (PA), an endocrine emergency caused by acute hemorrhage and/or infarction in the pituitary gland, represents an acute clinical syndrome presenting as a severe headache and decreased vision, ophthalmoplegia, and even altered consciousness [1,2,3]. PA may lead to hormonal deficiencies including life-threatening adrenal insufficiency and diabetes insipidus (DI), regardless of the presence of a prior pituitary mass [4,5,6,7,8,9]. PA in pregnancy has been exceptionally reported, but it remains of major importance to be adequately recognized and treated because it can lead to a fatal outcome for both the mother and fetus [10,11,12,13,14].

Generally, PA occurs in from 2 to 12% of patients with pituitary adenomas [15]; for instance, a prevalence of 8% is reported for non-functioning macroadenomas [16], and a prevalence of 6.8% is reported in lactotroph pituitary neuroendocrine tumors (PitNETs) [17]. The true prevalence, however, is still unknown [18,19]. Grand’Maison et al. estimated PA prevalence in pregnancy and the postpartum period (PP) to be 1 per 10,000 term pregnancies [20].

Outside pregnancy and lactation, PA is associated with a number of risk factors including cardiac surgery and other major surgeries, head trauma, arterial hypertension, coagulation disorders, anticoagulant treatment, pituitary stimulation tests, initiation or withdrawal of dopamine agonist treatment, radiotherapy, etc. [17,21,22,23].

During pregnancy, a series of changes that impact the pituitary gland occur. Physiological hormonal secretion from the placenta mediates an increase in the ovarian production of estrogen and progesterone. The surge in estrogen levels stimulates the pituitary gland and leads to the hypertrophy of lactotroph cells as well as hyperplasia up to 50% [1,24,25]. High levels of estrogens may also lead to hyperemia of the pituitary [26,27,28]. Furthermore, during pregnancy, there is an increase in pituitary volume, starting from the first weeks of pregnancy, but an increase in pituitary tumor size has also been noted, especially in lactotroph tumors, during the preparation period for lactation [24,29,30,31,32,33,34,35,36,37,38]. Another contributing factor is the pro-thrombotic state accompanying pregnancy [39].

In pregnant women, PA most commonly occurs in patients with previous pituitary adenomas as a result of their gestational hypertrophy. Lactotroph tumors, in particular, may enlarge during pregnancy, increasing the risk of PA and associated neuro-ophthalmological consequences such as the compression of the optic chiasm or cranial nerves. Often, PA in pregnancy is identified in patients with an undiagnosed PitNET as the first manifestation of the disease. However, due to the physiological gestational increase in pituitary volume, PA may occur in patients without any underlying hypophyseal mass [40,41,42].

Due to its clinical presentation with acute headache as the core manifestation, PA can be mistaken for a series of other serious conditions for which pregnancy may also increase risk, such as preeclampsia, cerebral venous thrombosis, subarachnoid hemorrhage, and arterial dissection. Other conditions that may present with headache and should be included in the differential diagnosis include meningitis, idiopathic intracranial hypertension, and migraine [43,44,45,46]. PA should also be differentiated from hyperemeis gravidarum due to the possible presentation with nausea and severe vomiting [47].

The diagnosis of PA in pregnancy is confirmed by magnetic resonance imaging (MRI) [19,22,48,49], which identifies intrasellar hemorrhage [50]. In addition to confirming PA, MRI is an important tool for the differential diagnosis of neurologic disorders associated with pregnancy [50,51].

Generally, the initial approach of patients with PA is conservative, aiming to ensure fluid and electrolyte balance and to restore glucocorticoids replacement. Steroids are also indicated for the prevention of cerebral edema. The next step is the decision to either continue conservative treatment, with the possibility of administering dopamine agonists (DA) or to undergo surgery [14,19,52,53,54]. Patients with prolactinoma taking DAs should stop the medication when pregnancy is confirmed. In particular cases, such as invasive adenoma or continuous tumor growth, DAs should be resumed [55,56,57,58].

Concerning PA in pregnancy, the conservative approach is often preferred. Due to the risk for the mother and fetus, surgical management during pregnancy is reserved for patients with deteriorating consciousness or severe neuro-ophthalmological deficits [57,59,60,61]. When surgical management is chosen, the second trimester and early third trimester are preferred [59,62], with no preference regarding the anesthetic approach [63].

### Aim

Our purpose is to provide new insights for AP in pregnancy and the PP period by covering different aspects from presentation to outcome.

## 2. Methods

This is a narrative review of the English medical literature regarding PA in pregnancy and PP, using a PubMed-based search with the following keywords: “pituitary apoplexy” and “gestational” or, alternatively, “pregnancy” or “postpartum”. The inclusion criteria consisted of clinically relevant original studies with a publication date between January 2012 and December 2022. We excluded other etiologies of PA.

**Figure 1:**
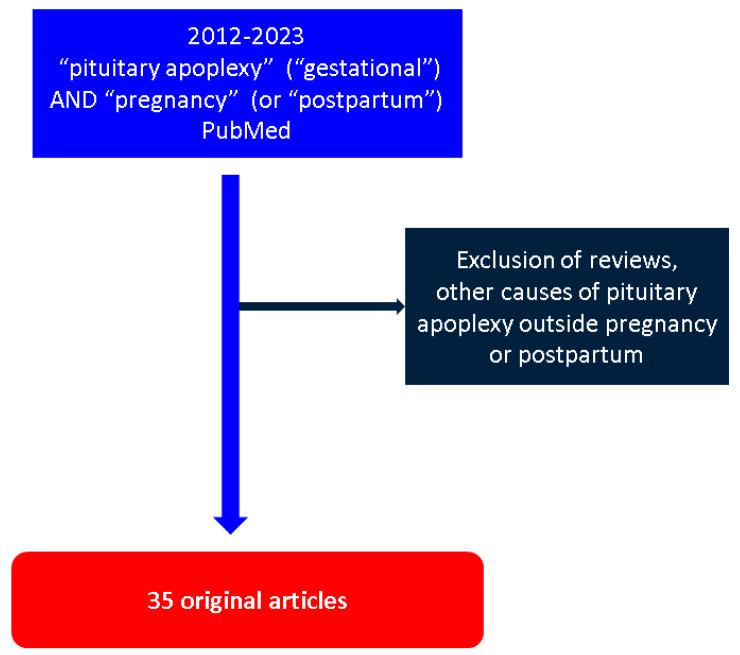
Flowchart diagram according to our methodology.

Overall, we identified and analyzed 35 papers; among them, 30 original studies (including case reports) addressed subjects with PA in pregnancy, and 6 case reports recorded PA during PP (notably, 1 study, which includes 2 previously unreported individuals with PA in pregnancy and one during PP, is common to both sections). There was a total of 50 patients analyzed from the published data, including 44 patients with PA in pregnancy and 6 with PA during PP (Figure 1).

## 3. Results

### 3.1. Sample–Case Series Study

Findings regarding PA in pregnancy are summarized in Table 1 and Table 2.

We identified three observational studies, of which two were retrospective [77,82] and one was not [78]. Galvão et al. [77] published a retrospective analysis investigating the consequences of pregnancy in patients with a previous diagnostic of a prolactinoma. Overall, 33 out of the 35 women had lactotroph PitNETs diagnosed before pregnancy. The majority of the patients stopped medical treatment within the 8th week of gestation (WG), and no cases of malformations were reported. No significant progression of the underlying disease was observed during pregnancy. In total, 2 out of the 35 patients developed PA in pregnancy (28 WG and 25 WG). The first patient was treated conservatively, and the second one was treated surgically. Both women were admitted for headaches and visual disturbances. They had no previously diagnostic of a PitNET, and, thus, they did not receive DAs before pregnancy. The surgically treated patient developed hypothyroidism and DI [77].

The risk of pituitary adenoma enlargement is higher during pregnancy. Barraud S et al. [82] published a retrospective study also following pregnancies in females with lactotroph PitNETs and the associated risk of tumor growth. Overall, 85 pregnancies (46/85 were macroprolactinomas), in women who were treated with DAs before pregnancy, were included. Adenoma growth and symptomatic tumor progression occurred in 19.6% of cases. In total, 3/85 women had PA in pregnancy; none was under DAs. Emergency trans-sphenoidal surgery (TSS) was performed in 2/3 females with PA due to vision anomalies (within the 4th month and 36th WG) [82]

Lambert et al. [78] published a prospective study on 71 pregnant subjects with pituitary tumors (49/71 macroprolactinoma, 16/71 non-functioning adenomas, 3/71 somatotropinoma, and 3/71-corticotropinoma). In the study, 2/71 subjects developed PA (one with macroprolactinoma and the other with non-functioning pituitary adenoma), and they were conservatively managed [78].

The largest cohort of consecutive PAs in pregnancy contains from three to five individuals/series [20,81,88,90]. Grand’Maison et al. [20] introduced 4 PAs during pregnancy and PP (one case was excluded due to previous publication by Couture et al. [64] in 2012, and the fourth case is presented in the section regarding PA in PP and summarized in Table 3) [20,64]. The remaining 2/4 cases, two females of 33 and 30 years of age, developed PA at 39 WG and 20 WG, respectively, while being admitted for headache and visual disturbances. One of the patients had a history of prolactinoma and was treated with cabergoline before pregnancy; DA was stopped within the first trimester, but it was restarted. One subject associated high blood pressure and preeclampsia. Patients were treated conservatively, and they delivered at term two healthy newborns [20].

Kato et al. [88] reported three PAs in pregnancy (median maternal age of 28 years). One of the women was known to have lactotroph PitNET before pregnancy. The median gestational age at the beginning of symptoms (headache and visual field defects) was 31 WG. The management was similar: a conservative approach amid pregnancy in 100% of cases; the babies were delivered by caesarean section; after birth, all subjects suffered TSS. The postoperative pathological examination confirmed 2/3 lactotroph PitNETs and one plurihormonal PitNET (lactotroph and gonadotroph). After 3 months of follow-up, the patients had no signs of hypopituitarism [88]

Kuhn et al. [90] identified five PAs in pregnancy (median maternal age of 26 years) with a pre-gestational confirmation of lactotroph PitNETs. The median gestational age of PA was 26 WG. Initially, conservative therapy was chosen, but one female underwent TSS during pregnancy and another after delivery. As hormonal complications, we mention DI (1/3), and adrenal insufficiency (1/3) [90].

Another series of three subjects was introduced by Jemel et al. [81]. The median maternal age was 32 years, and one woman was diagnosed with a pituitary adenoma before pregnancy. The median gestational age at the beginning of symptoms was 27 WG. The management was different: while 2/3 had TSS, 1/3 had conservative therapy [81].

Overall, most data are provided from case reports rather than original studies specifically addressing pregnancies complicated with PA. The cited studies are observational and retrospective. The majority of pituitary masses, if their type is known, were prolactinomas. The enlargement of the mass is often reflected in the clinical presentation, specifically with headache (followed by a heterogeneous spectrum of visual anomalies of different degrees of severity), which is a common finding with the data we currently have on other types of PA outside gestation. The specific medication for prolactinomas, as cabergoline or bromocriptine, was stopped within the first weeks of pregnancy confirmation as generally recommended. Most studies enrolled subjects within their third decade of life [20,64,65,66,67,68,69,70,71,72,73,74,75,76,77,78,79,80,81,82,83,84,85,86,87,88,89,90,91,92].

### 3.2. Patients’ Characteristics: Pregnancy Features

The age at the presentation of gestational PA ranged between 21 years [90] and 41 years [69] with an average age of 27.76 years. In total, 21/44 patients presented with signs and symptoms of PA during the 3rd trimester [20,70,71,72,73,74,76,77,79,80,81,82,83,84,85,86,87,88,89,90,91], and 1 female was admitted for PA during the first trimester [65] (the others presented during the second trimester). The mean WG at presentation was 26.38, the earliest at 10 WG [65] and the latest at 39 WG [20]. Most subjects (N = 9) were prim gravidae [20,65,70,73,76,80,81,87,89]. Moreover, one patient was nulliparous [85], one was primipara [77], one was P1 [88], two were G2 [81,92], two were G3 [86,90], two were G5 [74,85], one patient was G6P3A2 [20], and one was G6P4 [75]. A total of seven females had previous deliveries [20,74,75,81,84,88,90].

In total, 30/44 patients with data available regarding the method of delivery, 19/30 patients underwent a C-section (CS) [64,66,67,70,71,74,76,78,80,82,85,86,87,88,89,90,91], and 11/30 women underwent vaginal delivery (VD) [20,65,69,73,83,84,89,90]. In addition, one subject underwent CS in both of her pregnancies [86]. Furthermore, 32/44 patients with data available regarding WG at delivery, 25 underwent full-term delivery [20,64,65,66,67,69,71,73,74,76,79,80,81,83,84,85,87,88,90,91], and 7 females are mentioned to have delivered preterm babies [70,82,86,88,89]. Notably, one subject delivered preterm twins at 28 WG who died 7 days after birth [89].

### 3.3. Onset of PA in Pregnancy: Focus on Clinical Panel

The most frequent symptom that was found in almost all patients was headache; only 2/44 females were headache free [88,90]. In the study of Barraud et al.’s [82] patients, specific symptoms were unavailable [82]. Headache was described as “severe” [20,70,71,73,74,75,76,80,81,90,91], “unbearable” [90], or with acute onset [20,71,81]. Similarly, a pulsating character was reported by Geissler et al. [86]. A lack of response to anti-pain treatment was also mentioned in two persons [79,91]. Chan et al., however, described a mild headache [84]. Localization of the pain varied: fronto-parietal, fronto-orbital, temporal, and occipital were all present, as well as irradiation to the forehead [70,74,81,86,87,92]. Headache was often accompanied by nausea and vomiting [20,64,74,81,83,86,87,91,92]. Projectile vomiting was also observed [92].

Other frequent symptoms were visual symptoms. Visual disturbances decreased visual acuity [74,77,81,84,92], varying from visual blur [64,74,77,81] to the transient loss of vision [74] and visual field defects [20,68,69,71,77,79,80,82,85,88,90], including hemianopia [66,69,75,88,90] and unilateral vision loss [68,71,80,90]. Repetitive flashes of light were also reported [86]. In terms of cranial nerve palsies, oculomotor nerve palsies, manifesting as ptosis [92]; anisocoria [83,84]; and diplopia [75] were observed. Another form of presentation was transient DI as an initial symptom [90]; moreover, DI accompanied headache, nausea, vomiting, and anisocoria [83]. Signs of meningism such as photophobia [70,75,90] and neck stiffness [20] were reported too. Interestingly, in addition to headache and visual disturbances, a 30-year-old patient presented at 37 WG with numbness and weakness of the left side of the body. The author hypothesized that vasospasm of the intracavernous carotid artery was the probable cause of this phenomenon. The patient was treated with low-molecular-weight heparin. Following conservative management with hydrocortisone, symptoms resolved, with the exception of blurred vision and persistent hypocortisolism [74].

Even though most patients suffered a single episode of PA in pregnancy, Geissler et al. reported a repeated PA episode during two of her pregnancies. The patient presented with similar symptoms both times, with headache and visual disturbances at 34 WG and 32 WG, respectively. She also suffered from gestational diabetes mellitus (DM) requiring insulin therapy during both of her pregnancies. PA was conservatively approached each time. She delivered healthy babies by CS at 36 and 34 WG but was unable to breastfeed [86].

The diagnosis of PA was established starting from clinical presentation, as mentioned. A good multidisciplinary collaboration is required in this circumstance. The hormonal panel is classical for newly onset hypopituitarism. It investigates each line of pituitary hormones, mostly according to baseline blood assessment rather than using dynamic tests during pregnancy. In addition to endocrine confirmation of central hypothyroidism, adrenal insufficiency, and, in some cases, diabetes insipidus, the diagnosis also includes the imaging scans that show distinct features of apoplexy, tumor remnants or even intact areas of the pituitary gland [20,64,65,66,67,68,69,70,71,72,73,74,75,76,77,78,79,80,81,82,83,84,85,86,87,88,89,90,91,92].

### 3.4. Risk Factors for Developing PA in Pregnancy

Additional risk factors that have been identified are summarized in Table 1 and Table 2 and include treatment with DAs (15/44), gestational DM (N = 2), type 1 DM (N = 1), and acute COVID-19 infection [20,84,86,89,90]; moreover, the case studied by Grand’Maison et al. [20] suffered from both gestational DM and preeclampsia during previous pregnancies [20]. Overall, 15/44 females had previous treatment with DAs, as follows: 11/15 persons underwent cabergoline treatment [20,69,73,76,81,85,89,90], 4/15 used bromocriptine [68,71,73,90], and one woman was under terguride [88]. De Ycaza et al. [73] introduced a subject switching from bromocriptine to cabergoline due to side effects; when pregnancy was confirmed, DA was stopped [73].

Another risk factor was anticoagulant therapy. For example, Watson V et al. [74] reported the case of a 30-year-old woman, with an undiagnosed pituitary adenoma, who underwent prophylactic treatment with low-molecular-weight heparin throughout pregnancy. She displayed severe headache and visual disturbance at 37 WG. A pituitary hemorrhage was confirmed by MRI. The patient received conservative treatment with hydrocortisone and delivered the baby at term by CS. On discharge, she was offered oral hydrocortisone for persistent hypocortisolism [74].

### 3.5. Differentiating PA in Pregnancy from Other Entities

The differential diagnosis of PA in pregnancy first starts from headache, as is this case with, for example, eclampsia. The importance of differentiating between these conditions is highlighted by Sedai et al. [92]. In their report, a 40-year-old woman presented at 21 WG with headache, projectile vomiting, ptosis, decreased visual acuity, and altered consciousness. The patient received conservative management for eclampsia at first, but the progression of neurological deficits was consistent with further PA expansion of a previously undiagnosed PitNET. A craniotomy for tumor resection and hematoma evacuation was performed with a fatal outcome in the second postoperative day [92]. This case further emphasizes the importance of adequate management in PA. With regard to PA-associated meningism in terms of photophobia and neck stiffness, a differentiation from meningitis is necessary in these cases [20,70,75,90].

### 3.6. Conservative Management of PA Amid Pregnancy

Most patients (N = 29) received conservative management that included glucocorticoid supplementation with hydrocortisone or dexamethasone (1 case), thyroid substitution (in 3 cases) in addition to DAs; the cases with DI required desmopressin [20,64,65,67,68,73,74,76,77,78,79,81,83,84,86,87,88,89,90,91]. Kanneganti et al. [87] reported a 26-year-old primigravida at 37 WG who was free of previous medical problems and developed headache and visual disturbance in pregnancy. A pituitary MRI scan showed an enlarged pituitary gland with optic chiasma compression. The patient was treated conservatively with hydrocortisone. She gave birth a week later by cesarean section [87].

Cases with prolactinomas developing PA in pregnancy seem the most frequent with regard to the type of PitNET. Chegour et al. [68] reported PA in a 29-year-old woman with a macroprolactinoma unconfirmed before pregnancy, who received treatment with bromocriptine for hyperprolactinemia of uninvestigated etiology. At 19 WG, she presented with headache and visual disturbances due to PA, and she was conservatively managed with cabergoline leading to the complete regression of the visual symptoms [68]. Another interesting case history was reported by Couture et al. [64]: a 37-year-old female with a known lactotroph PitNET larger than 1 cm was treated with cabergoline before pregnancy. At 16 WG, she presented with headache, nausea, vomiting, and blurred vision, and MRI confirmed PA. Cabergoline treatment was resumed, resulting in regression of the pituitary mass after 5 weeks. Her pregnancy ended successfully at 38 WG with delivery by CS [64]. Annamalai et al. [76] reported a 25-year-old individual with a known macroprolactinoma treated with cabergoline; at 37 WG, she complained of headache, and MRI confirmed PA. She was offered hydrocortisone and resumed cabergoline. Two days after admission, the patient delivered a healthy baby by cesarean section. After 4 months of follow-up, the complete resolution of the pituitary adenoma was registered [76]. De Ycaza et al. [73] introduced a young female with a known macroprolactinoma treated with cabergoline that experienced headache at 28 WG confirmed with MRI as being PA. Cabergoline was resumed, and then she gave birth vaginally at term; 1 year later, the tumor was less than 1 cm, and substitution with hydrocortisone was stopped [73]. Additionally, Janssen et al. [65] reported a woman with a lactotroph PitNET larger than 1 cm who received treatment with bromocriptine until pregnancy was confirmed. At 10 WG, she developed PA, and bromocriptine was resumed in association with hydrocortisone and L-thyroxine replacement. She gave birth vaginally at 40 WG [65].

### 3.7. Surgical Management in PA Amid Pregnancy

We have data concerning 22 females who underwent TSS for tumor resection, either as an initial measure (N = 10) [66,69,70,71,75,77,82,85,90], following conservative treatment (N = 4) [67,79,81], or electively after birth (N = 8) [80,82,84,88,90]. The endoscopic surgery was necessary in cases associated with acute nerve compression. TSS was performed as an initial measure or following conservative treatment in selected cases due to the persistence or worsening of visual defects [69,71,75,77,79,81,82,85,90] or the deterioration of their neurological condition [77].

Hayes et al. [69] reported a case of pituitary hemorrhage and compression of the optic nerve and chiasma. After corticosteroid treatment, the patient underwent TSS due to visual decline. She vaginally delivered a healthy boy at term. No hormonal deficits were detected, and, at 14 months after birth, the patient remained well [69]. Oguz et al. [85] reported a 26-year-old female diagnosed with prolactinoma 2 years prior to pregnancy. She presented at 22 WG with headache, nausea, and visual disturbance. After 8 days of admission, TSS was performed due to the persistence of visual symptoms. She delivered in good condition at 37 WG. Eight months after delivery, she was still treated with levothyroxine [85]. O’Neal et al. [79] reported a case of undiagnosed pituitary microadenoma in which the female had headache and visual disturbances at 29 WG. MRI showed an expanded pituitary with compression of the optic chiasma. Two days after admission she underwent TSS. She delivered at term a healthy boy. She also developed DI after surgery [79]. Abraham et al. [75] reported a spontaneous PA in pregnancy with sensory loss. The patient, a 32-year-old, developed headache, photophobia, and right-sided numbness at 23 WG. Emergency surgery was performed with decompression of the optic nerve. She developed DI on the second postoperative day, requiring desmopressin [75]. In another case, a 27-year-old female received the first diagnosis of prolactinoma at 19 WG and bromocriptine was administrated. At 36 WG, she had headache and acute vision loss in the left eye with MRI confirmation of a hemorrhagic pituitary mass of 2.1 cm maximum diameter with optic chiasm compression. The patient underwent TSS with good postoperative course. A cesarian section was performed, and a healthy baby was born. At follow-up, MRI showed the complete resolution of tumor [71].

In other studies, pituitary surgery was performed PP (N = 9) [70,80,82,84,88,90], during the second trimester (N = 9) [66,67,69,75,77,81,82,85], and during the third trimester (N = 4) [71,79,82,90]. As mentioned, a craniotomy was the alternative to TSS when a fatal outcome might be found [92].

### 3.8. PitNET Analysis

Overall, 18/44 patients had a pituitary adenoma undiagnosed before pregnancy, and 21 patients had a known pituitary adenoma (please see Table 1 and Table 2). However, three studies included only patients with known pituitary adenomas, and some of them further developed PA [77,78,82]. Notably, one female was diagnosed during pregnancy with a pituitary adenoma and developed PA later during pregnancy [71]. Even though almost all patients had pituitary adenomas, one patient developed PA due to pituitary hyperplasia, without adenoma [20], and in another case, no pituitary adenoma was found at all [75]. Most pituitary adenomas were lactotroph PitNETs (N = 26), while one patient had a lacto-gonadotroph PitNET [88], and a non-functional adenoma was present in three subjects [66,78,81]. Out of lactotroph tumors, the majority (N = 16) were larger than 1 cm, while one prolactinoma was giant, measuring 4.5 cm maximum diameter [89].

### 3.9. Outcome of PA in Pregnancy

Most patients experienced the post-PA resolution of symptoms. Seven patients experienced a decrease in tumor size, and four had no residual tumor [64,67,68,85]. Following PA, some patients developed hormonal deficits, including corticotropic deficiency [65,74,83,84,89,90], central hypothyroidism [70,77,84,85,89], hypogonadism [84], persistent DI [66,77,79,83,90], or transient DI [71,75], while at least eight patients had normal pituitary function [20,67,69,75,76,80,86].

Moreover, Ye et al. [91] published a case of PA associated with extra-pontine myelinolysis in pregnancy at 32 WG with no previously known pituitary adenoma and that presented with vomiting. The laboratory analysis showed hyponatremia, so the patient received sodium repletion. She developed aphasia and hemiplegia the next day. MRI showed PA and abnormal signals in some areas that suggested extra-pontine myelinolysis. The patient received treatment with hydrocortisone and levothyroxine. She gave birth at 38 WG (CS) to a healthy baby [91].

A fatal outcome of the newborn was reported by Khaldi et al. on a case of a giant prolactinoma complicated with PA in pregnancy. The subject was treated with cabergoline and surgery and presented at 22 WG with symptoms of PA. She received treatment with bromocriptine and hydrocortisone. She developed corticotropic and thyrotrophic insufficiency. Unfortunately, she gave birth prematurely at 28 WG to twins who died on the 7th day of life [89]. In another study, a fatal maternal outcome was reported following a craniotomy for PA at 21 WG [92].

Regarding breastfeeding in females who experienced PA in pregnancy, we have the data on three patients who were unable to breastfeed [86,90,91], while one patient was able to breastfeed for only 2 weeks [69]. Two patients were able to have an uneventful second pregnancy following PA amid previous gestation [20,73].

### 3.10. PA during PP

Six cases of PA in PP state were identified during our search [20,93,94,95,96,97]. The findings are summarized in (Table 3; one study being also cited in Table 1 and Table 2—please see reference [20]).

**Table 3:** Characteristics, clinical presentation, management, and outcome of patients with pituitary apoplexy during PP [20,93,94,95,96,97].

Reference (Name, Number, and Year of Publication)	Type of Study	Population	Gravidity and Parity	Days at PP on Presentation	Clinical Presentation	Preexisting Pituitary Lesion	Treatment	Delivery	Maternal Outcome	Other
Mathur [93] 2014	Case report	34-year-old female	G2P1	5 min PP	Persistent headache for 48 h following CS with spinal anesthesia (with onset 5 min after delivery) Nausea Transient DI	No pituitary adenoma	Conservative management with oral hydrocortisone	LB by CS	Normal pituitary function Pituitary enlargement adjacent to the chiasma	Ten days after PA the patient developed RCVS
Grand’Maison [20] 2015	Case series	Four cases of PA related to pregnancy, of which one, a 40-year-old female, was of PA during PP	G4P1A3	6 h PP	Headache and unable to lactate	No pituitary mass	Conservative management with cortisol supplementation	LB at 36 WG by VD with forceps	Adrenal insufficiency for 7 months post-partum, GH deficiency, and atrophic pituitary gland	The patient also suffered from type 1 DM and primary hypothyroidism
Raina [94] 2015	Case report	27-year-old female	G2	2 days PP	Headache Blurred vision Ptosis Diplopia	No pituitary adenoma	Conservative management with hydrocortisone 50 mg 6 times hourly Thyroid hormone replacement therapy	LB by VD (home-conducted)	Complete recovery and normalized thyroid function	The patient suffered from PP hemorrhage
Dias [95] 2021	Case report	37-year-old female	G2	12 days PP	Occipital headache Nausea Vomiting Fever	No pituitary adenoma	Conservative (NSAIDs and IV fluids)	LB at term by CS	Hypothyroidism	
Hoang [96] 2022	Case report	34-year-old female	primigravida	2 days PP	Right facial paralysis Headache Eye pain Blurred vision	No pituitary adenoma	Conservative	LB at 38th by CS	Complete recovery Normal pituitary function	The patient also suffered from a subdural hematoma
Pop [97] 2022	Case report	26-year-old female	primigravida	48 h PP	Headache Nausea Photophobia 3rd cranial nerve palsy: left ptosis and anisocoria Polyuria and polydipsia	NFPA of 3.3 × 1.05 × 1.55 cm without compression on the optic chiasma	Initial conservative management with dexamethasone and LT4 TSS	LB at 40th by CS	Complete neurological recovery at 2 years follow-up: HRT for panhypopituitarism	

Abbreviations: A = abortion; NSAID = nonsteroidal anti-inflammatory drug; CS = cesarian section; DI = diabetes insipidus; DM = diabetes mellitus; G = gesta; GH = growth hormone; HRT = hormone replacement treatment; IV = intravenous; LB = live birth; NFPA = non-functioning pituitary adenoma; P = para; PA = pituitary apoplexy; PP = postpartum; RCVS = reversible cerebral vasoconstrictive syndrome; TSS = trans-sphenoidal surgery; VD = vaginal delivery; WG = weeks of gestation.

The mean age of patients who suffered PA in PP was 33 years. Three patients were in their second pregnancies, two patients were prim gravidae [93,95,96,97], and one female was G4P1A3 [20]. Overall, 4/6 individuals had CS, 2/6 had VD (including one that was home-conducted) [20,93,94,95,96,97]. The timing of symptoms was as early as 5 min after delivery [93] and as late as 12 days after delivery [95], including 3/6 females with PA 2 days following delivery [94,96,97].

All patients presented headache as the main symptom, which was described as severe (2/6); throbbing/pulsatile (2/6); either frontal (1/6), frontotemporal (1/6), or occipital (1/6); and accompanied by eye pain (1/6), nausea (3/6), and vomiting (1/6). In the case of Mathur et al.’s patient, headache persisted over the course of 48 h without remission after treatment with paracetamol [93]. Visual symptoms (N = 3) included a decrease in visual acuity, diplopia, ptosis, and anisocoria. Photophobia (N = 1), fever (N = 1), polyuria and polydipsia (N = 2), and the inability to lactate (N = 1) were also identified. Raina S et al. [94] presented a case of PA in PP associated with isolated third cranial nerve palsy; the patient had a history of PP hemorrhage following a full-term home-conducted vaginal delivery. On the second day of admission, she complained of blurred vision, headache, and diplopia. Ptosis on the right side was also noted. MRI established the diagnosis of PA. She started thyroid hormone replacement therapy along with oral hydrocortisone. During follow-up, the subject had a full recovery with the normalization of thyroid function [94].

In one study, 5/6 subjects had no underlying pituitary adenoma [20,93,94,95,96]. Pop et al. reported a non-functioning large pituitary adenoma of 3.3 × 1.05 × 1.55 cm that was undiagnosed before pregnancy [97].

Two patients associated additional risk factors: type 1 diabetes [20] and postpartum hemorrhage [94]. A co-morbidity in terms of subdural hematoma is reported by Hoang et al. [96], in which a 34-year-old subject experienced PA in PP and subdural hematoma following epidural anesthesia. She delivered by CS at 38 WG, and 2 days after delivery, she showed signs of right facial paralysis, which was associated with headaches, eye pain, and blurred vision. MRI confirmed PA and a left frontal subdural hematoma. PA was conservatively approached followed by a full recovery within 1 year [96]. Another incidental event is reversible cerebral vasoconstrictive syndrome (RCVS). Mathur et al. [93] reported a 34-year-old female who developed PA after an emergency CS under spinal anesthesia. She had severe PP headache and neurologic deficits. MRI showed PA. DI developed after 48 h. She was managed conservatively with oral hydrocortisone in order to prevent secondary adrenal insufficiency. Ten days following PA, the persistent headache led to the identification of a new subarachnoid hemorrhage on MRI; she was further confirmed with reversible cerebral vasoconstrictive syndrome. Twenty months after the event, she did not require any hormone replacement therapy, but the MRI showed the enlargement of the pituitary bordering the optic chiasm [93].

Overall, 5/6 patients were managed conservatively [20,93,94,95,96], and 1/6 underwent TSS [97]. The conservative treatment consisted of vital sign monitoring, airway support, nutritional support, glucocorticoids supplementation, fluid, and electrolyte replacement, as well as non-steroidal anti-inflammatory drugs [20,93,94,95,96]. The patient of Pop et al. underwent TSS following initial conservative management due to deteriorating consciousness [97].

Pituitary function recovered and remained normal at the latest follow-up in three of the six patients [93,94,96], while the other three subjects required therapy for hypopituitarism [20,95,97].

In the case of Mathur et al.’s patient, a differential diagnosis of headache included multiple conditions: this is a 34-year-old female who underwent CS under spinal anesthesia and received a bolus of oxytocin at delivery. The patient complained of persistent headache over the course of 48 h after delivery. MRI scans were performed and showed pituitary hemorrhage. Both spinal anesthesia and oxytocin bolus may cause headache in PP, therefore complicating the differential diagnosis of PA [93].

Overall, a negative outcome concerning the newborn was reported in one twin pregnancy within the seventh day after birth [89]. PA in pregnancy caused premature babies in some cases [70,82,86,88]. Peripartum data suggested PA onset a few hours following domestic birth (after 36 weeks of gestation) [20] or a few days [94]. The confirmation of PA in pregnant females required an emergency cesarean section due to visual field progressive anomalies [82]. However, since PA is typically diagnosed in advanced pregnancies, data on healthy living are specifically provided in 29 reports [20,69,70,71,73,74,76,77,79,81,82,83,84,85,86,87,88,89,90,92,93,94,95,96,97,98].

## 4. Discussions

**Figure 2:**
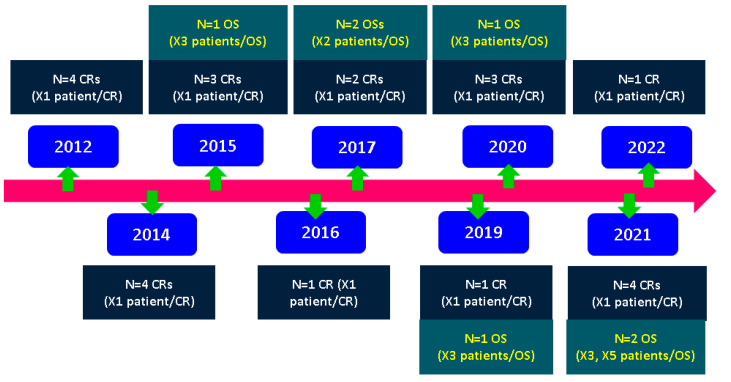
Timeline diagram of studies regarding PA in pregnancy according to our analysis [20,64,65,66,67,68,69,70,71,72,73,74,75,76,77,78,79,80,81,82,83,84,85,86,87,88,89,90,91,92,93,94,95,96,97]. Abbreviations: CR = case report; N = number of patients; OS = original study.

Our case-sample-based analysis followed 35 original publications: 7 studies (selected cases on PA from larger cohorts that included 22 women), and 28 case reports (1 patient/article, N = 28); thus, a total of 50 subjects were considered (44 with PA in pregnancy and 6 with PA diagnosed after delivery). We noticed that the original studies were of small sample sizes (the highest number of females with the actual diagnosis of PA was 5), and the studies addressed different issues of PitNETs outside PA (the largest cohorts consisted of 35, 46, and 71 patients with PitNETs). Notably, we used the terms of “case series” or “study” in a table according to the original publication, but our final report, as introduced below, takes into consideration 28 case reports (1 female/paper) and 7 non-case reports (2–5 females/paper) that specifically refer to PA (Figure 2).

### 4.1. Integrating PA in Pregnancy and PP to the Larger Frame of PAs

The growth of pituitary tumors, especially lactotroph tumors, during pregnancy, as well as pituitary hypertrophy, increases the risk of PA in gestation. The low number of cases found between 2012 and 2022, however, suggests the rarity of the disease. The clinical presentation of patients with PA in pregnancy and PP is similar to the clinical presentation of non-pregnant patients with PA with sudden and severe headache; nausea and vomiting; visual disturbances including a decrease in visual acuity; and signs and symptoms of cranial nerve palsies such as visual field defects, ptosis, anisocoria, and diplopia [14,39,98,99,100,101,102]. Presentation with hypocortisolism occurred in one of the patients with PA in pregnancy [91].

We also observed DI as an initial presentation in patients with PA during pregnancy and PP [83,93,97]. The manifestation of DI during pregnancy ranges from the exacerbation of pre-existing central or nephrogenic DI to pregnancy-induced transient DI due to the increased metabolism of the antidiuretic hormone vasopressin (AVP) by placental vasopressinase [12,99,103,104,105].

We mentioned that many patients present during pregnancy or PP with PA as the initial symptom of a previously undiagnosed PitNET. In patients with known pituitary tumors, the most frequent type was lactotroph PitNET. Additional risk factors are gestational and type 1 DM, while pre-gestation treatment with DA was discontinued at the moment of pregnancy confirmation. The majority of prolactinomas were macroadenomas; thus, it could be hypothesized that the tumor size may increase the risk of PA in pregnancy.

The management of PA is similar to that of non-pregnant patients. Most patients were treated conservatively, while surgery was reserved for cases with persistent and evolving visual disturbances or altered consciousness. The TSS was preferred in all postpartum cases except for one [92].

Generally, the maternal–fetal outcomes are favorable. Hormonal deficits are relatively frequent, and they include hypocortisolism, hypothyroidism, and sometimes hypogonadism or growth hormone deficits. One noticeable postoperative complication observed in patients with PA in pregnancy that underwent surgery was DI, either transient or persistent [66,71,77,79]. No case of neonatal abnormalities and congenital malformation were observed. We still do not have long-term surveillance studies of children born from mothers who experienced PA in pregnancy or PP.

As seen outside pregnancy, the differential diagnosis of headache is crucial in establishing an adequate diagnosis. PA in pregnancy may mimic a series of conditions including eclampsia [92], meningitis due to photophobia [70,75,90], and nuchal rigidity [20]. In the PP, a differential diagnosis includes anesthesia and ocytocin bolus [93]. Another condition associated with headache was RCVS (N = 1) [93]. RCVS is a condition accompanied by the constriction of cerebral arteries, manifesting with headaches and possible neurological deficits. Its frequency is higher during PP. Its resolution is spontaneous, but it may lead to subarachnoid hemorrhage and even hemorrhagic or ischemic strokes [98,106,107,108].

Due to the gravity of the aforementioned conditions, differential diagnoses and prompt and proper treatment are essential, as illustrated by one case with an initial misdiagnosis that led to a delay in identifying and treating PA and, finally, to exitus [92].

### 4.2. PA in Pregnancy versus Postpartum

As expected, we found more cases of PA in pregnancy than postpartum (44 versus 6 individuals). PA during pregnancy and PP share a similar clinical presentation with headaches of similar patterns and localizations accompanied by not only nausea and vomiting but also visual symptoms including a decrease in visual acuity, visual field defects, diplopia, and anisocoria. Ptosis was observed only in one patient suffering from PA during PP [97], and it was observed in pregnancy-associated PA. Further clinical similarities include photophobia, polyuria, and polydipsia and lack/difficulties of lactation [20,86,90,91]. In terms of underlying conditions, prolactinomas are most important for PA in pregnancy, while 5/6 patients suffering from PA during PP had no pituitary adenoma; however, the analysis remains at case report levels [20,93,94,95,96]. Similar risk factors such as type 1 diabetes are commonly listed too [20,90]. Most patients received conservative treatment both in pregnancy and PP. During pregnancy, TSS was performed due to the persistence or worsening of visual symptoms [69,71,75,77,79,81,82,85,90], while in PP, TSS (N = 1) was performed due to a decrease in consciousness [97]. Outcomes in these cases were similar. Notably, DI was reported only in relation to PA during gestation [66,71,77,79]. (Table 4).

### 4.3. Integrating PA Amid Other Endocrine Complications of Pregnancy

Generally, despite a low level of statistical evidence, PA in pregnancy remains a key element of the endocrine conditions that require particular intervention amid gestation, which involve the thyroid, adrenal, and pituitary glands [57,109,110,111]. Moreover, PA in pregnancy is one of the causes of acquired hypopituitarism in females during gestation [112]. For instance, Bichard et al. [83] reported the case of a 29-year-old woman developing headache, nausea, vomiting, and polyuria at 30 WG. MRI confirmed PA. She experienced panhypopituitarism, requiring hydrocortisone, levothyroxine, and desmopressin [83]. Another example was published by Pop et al. [97]: A 26-year-old female underwent CS. Forty-eight hours later, she complained of headache, photophobia, and nausea, and MRI confirmed a pituitary tumor of more than 3 cm, the largest diameter without compression, on the optic chiasma. On the 8th day after giving birth, she developed panhypopituitarism. Initially, she received conservative treatment, but due to deteriorating consciousness, surgical decompression was performed. At the 2-year follow-up, the patient remained on levothyroxine, prednisone, and estrogen–progestin replacement therapy [97]. Pregnancy-related hypophysitis is another cause of gestation-related pituitary insufficiency, and a recent retrospective analysis identified 148 of such published cases [112]. Additionally, Sheehan syndrome leads to hypopituitarism after a post-partum dramatic event, such as a hemorrhage, due to an obstetric event, and it should be differentiated from PA in PP [34,113,114,115]. Notably, PA in PP and Sheehan syndrome might be found in women who were otherwise healthy, thus displaying a low index of clinical suspicion [34,113,114,115].

### 4.4. COVID-19 Infection Associated with PA

Chan JL et al. [84] reported a case of a pregnant woman with PA suffering from SARS-CoV-2 infection. The patient presented at 38 WG with visual disturbance and headache. MRI showed a previously undiagnosed pituitary tumor with acute hemorrhage. She received conservative treatment with dexamethasone and gave birth to a healthy baby at 39 WG by VD. Two days later, TSS was performed. Two months further on, she still had hypothyroidism, hypogonadism, and hypocortisolism requiring hormonal substitution. It remains unclear whether the SARS-CoV-2 infection was a factor leading to PA or a mere coincidence [84]. However, we already know that COVID-19 infection is a new trigger for many conditions, during pregnancy or not, which are located at different organs and systems, and further evidence on coronavirus-associated PA is expected to be published [116,117,118,119,120].

## 5. Conclusions

PA in pregnancy is a rare, life-threatening condition. Most patients presenting with PA are primigravidae in the second or third trimester. Headache is the most frequent presentation, and its prompt distinction from other conditions associated with headache, such as preeclampsia and meningitis, is essential. The index of suspicion should be high, especially in patients with additional risk factors such as pre-gestation treatment with dopamine agonists, diabetes mellitus, anticoagulation therapy or large pituitary tumors. PA management is conservative in most cases, and it mainly includes corticosteroid substitution and dopamine agonists. The most frequent surgical indication is neuro-ophthalmological deterioration, although the actual risk of pituitary surgery during pregnancy remains unknown. PA in PP is exceptionally reported. To our knowledge, this sample–case series study is the largest of its kind that is meant to increase the awareness to the benefit of the maternal–fetal outcomes.

## Figures and Tables

**Table 1:** Characteristics, clinical presentation, management, and outcome of patients with PA in pregnancy. The studies are displayed starting with those from 2012 [20,64,65,66,67,68,69,70,71,72,73,74,75,76,77,78,79,80,81,82,83,84,85,86,87,88,89,90,91,92]. This table introduces the studied population, week of gestation, and clinical presentation, including the data on preexisting pituitary lesions.

Reference (Name, Number, and Year of Publication) and Type of Study	Population	WG on Presentation	Clinical Presentation	Preexisting Pituitary Lesion
Couture [64] 2012 Case report	37 y F	16 WG	Headache Nausea Vomiting Blurred vision	Lactotroph PitNET < 1 cm—diagnosed before pregnancy and treated with DA (bromocriptine switched to cabergoline) until pregnancy was confirmed
Jansssen [65] 2012 Case report	27 y F	10 WG (G1)	Headache Visual disturbance	Lactotroph PitNET > 1 cm—diagnosed before pregnancy and treated with DA (bromocriptine)
Kita [66] 2012 Case report	26 y F	26 WG	Headache Bitemporal hemianopsia	NFPA > 1 cm
Witek [67] 2012 Case report	26 y F	14 WG	Headache Visual field abnormalities	Lactotroph PitNET > 1 cm—diagnosed before pregnancy and treated with DA (bromocriptine)
Chegour [68] 2014 Case report	29 y F	19 WG	Headache Visual disturbances (unilateral vision loss)	Lactotroph PitNET > 1 cm—undiagnosed before pregnancy The patient received treatment with DA (bromocriptine) before pregnancy for hyperprolactinemia of uninvestigated etiology
Hayes [69] 2014 Case report	41 y F	18 WG	Headache Visual disturbances (visual field defects)	Pituitary adenoma—diagnosed before pregnancy (Lactotroph PitNET)
Piantanida [70] 2014 Case report	27 y F	35 WG (G1)	Headache Photophobia Bitemporal hemianopsia	Pituitary adenoma—undiagnosed before pregnancy
Tandon [71] 2014 Case report	27 y F	36 WG	Headache Unilateral vision loss	Lactotroph PitNET—diagnosed during pregnancy at 19 WG and treated with bromocriptine
Bedford [72] 2015 Case report	35 y F	NA	Headache	Pituitary macroadenoma
De Ycaza [73] 2015 Case report	26 y F	28 WG (G1)	Headache	Macroprolactinoma—diagnosed before pregnancy
Grand’Maison [20] 2015 Case series	4 F with PA #	Patient 1: 39 WG (G6P3A2) Patient 2: 20 WG (G1) Patient 4: G4P1A3	Patient 1: Headache, nausea, blurred vision, and neck stiffness Patient 2: Headache	Patient 1: Pituitary hyperplasia without preexisting lesion Patient 2: Lactotroph PitNET with regression after DA (cabergoline) treatment
Watson [74] 2015 Case report	30 y F	37 WG (G5P4)	Headache Visual disturbances Numbness and weakness of the left side of the body/transient left-sided facial numbness	Pituitary adenoma—undiagnosed before pregnancy
Abraham [75] 2016 Case report	32 y F	23 WG (G6P4)	Headache Photophobia Right-sided numbness Diplopia Superotemporal hemianopsia	No pituitary adenoma
Annamalai [76] 2017 Case report	25 y F	37 WG	Headache	Lactotroph PitNET—treated with DA for three months
Galvão [77] 2017 Retrospective, observational study	35 F ##	Patient 1: 28 WG (G1) Patient 2: 25 WG	Patient 1: Headache, blurred vision, and loss of consciousness Patient 2: Headache, blurred vision, and visual field defects	Patient 1: Lactotroph PitNET >1 cm Patient 2: Lactotroph PitNET
Lambert [78] 2017 Prospective, observational study	71 F ###	NA	Headache	Patient 1: Macroprolactinoma—diagnosed before pregnancy Patient 2: Non-functioning adenoma—diagnosed before pregnancy
O’Neal [79] 2017 Case report	27 y F	29 WG	Headache Visual field defects (2 days after start of conservative management)	Pituitary adenoma—undiagnosed before pregnancy
Bachmeier [80] 2019 Case report	30 y F	36 WG (G1)	Headaches Unilateral visual loss	Lactotroph PitNET—clinically asymptomatic and previously undiagnosed
Jemel [81] 2019 Case series	3 F with PA ####	Patient 1: 37 WG (G2P2A0) Patient 2: 22 WG (G1) Patient 3: 24 WG	Patient 1: Headache and blurred vision Patient 2: Headache, nausea, and vomiting Patient 3: Headache and visual disturbances	Patient 1: Pituitary adenoma—undiagnosed before pregnancy Patient 2: Non-secretory pituitary adenoma—the patient underwent treatment with DA for two years Patient 3: Pituitary macroadenoma—undiagnosed before pregnancy
Barraud [82] 2020 Retrospective, observational study	46 F ##### (3 F with PA)	Patient 1: NA Patient 2: 4th month of gestation Patient 3: 36 WG	Symptoms of PA, including visual field defects	Lactotroph PitNET ≥ 1 cm
Bichard [83] 2020 Case report	29 y F	30 WG	Headache Nausea Vomiting Anisocoria DI (Polydipsia 10 L/day and polyuria)	Pituitary adenoma—undiagnosed before pregnancy
Chan [84] 2020 Case report	28 y F	38 WG (G5P1)	Headache Decrease in visual acuity Anisocoria	Pituitary adenoma—undiagnosed before pregnancy
Oguz [85] 2020 Case report	26 y F	22 WG(G0)	Headache Nausea Visual disturbances (including visual field deficit)	Lactotroph pituitary macroadenoma
Geissler [86] 2021 Case report	27 y F	1st pregnancy: 34 WG (G3P0) 2nd pregnancy: 32 WG	Similar presentation for both pregnancies: Headache Visual disturbance (repetitive flashes of light)	Pituitary adenoma—undiagnosed before pregnancy
Kanneganti [87] 2021 Case report	26 y F	37 WG (G1)	Headache Visual disturbance Non-vertiginous giddiness Breast discharge	PA with optic chiasma compression
Kato [88] 2021 Case series	3 F with PA ######	Patient 1: 35 WG (P1) Patient 2: 32 WG Patient 3: 28 WG	Patient 1: Visual field defects (temporal hemianopia) Patient 2: Headache and visual field defects (temporal hemianopia) Patient 3: Headache	Patient 1: Lactotroph PitNET with compression on the optical chiasm (known before pregnancy) Patient 2: Lactotroph and gonadotroph PitNET with compression on the optical chiasm (undiagnosed before pregnancy) Patient 3: Lactotroph adenoma with compression on the optical chiasm (undiagnosed before pregnancy)
Khaldi [89] 2021 Case report	30 y F	22 WG	Headache Nausea Visual disturbance	Giant lactotroph PitNET—diagnosed before pregnancy and treated with DA and TSS with a 50% residual tumor
Kuhn [90] 2021 Case series	5 females with PA #######	Patient 1: 36 WG Patient 2: 26 WG Patient 3: 35 WG (G3) Patient 4: 16 WG Patient 5: 24 WG	Patient 1: Headache, visual impairment, bitemporal hemianopia, and photophobia Patient 2: Transient DI Patient 3: Headache and unilateral vision loss Patient 4: Headaches and visual field defects Patient 5: Headache	Patient 1: Lactotroph PitNET Patient 2: Lactotroph PitNET Patient 3: Lactotroph PitNET Patient 4: Lactotroph PitNET Patient 5: Lactotroph PitNET All patients were diagnosed with pituitary adenomas before pregnancy
Ye [91] 2021 Case report	24 y F	32 WG	Headache throughout pregnancy without remission under analgetic treatment Vomiting Dysarthria and hemiplegia after sodium supplementation Low-grade fever	Pituitary adenoma—undiagnosed before pregnancy
Sedai [92] 2022 Case report	40 y F	21 WG (G2P0A1L0)	Headache Projectile vomiting Ptosis Decreased visual acuity Altered consciousness	Pituitary adenoma—undiagnosed before pregnancy

Abbreviations: y = Years; F = female; NFPA = non-functioning pituitary adenoma. # PA during pregnancy or in the PP period with ages of 33 years (patient 1) and 30 years (patient 2); the case of patient 3 was previously published in 2012 and therefore not included; the case of patient 4 is summarized in Table 3. ## A total of 35 pregnant women with prolactinoma (2 women out of the 35 presented with PA during pregnancy at the age of 30 years—patient 1); NA (patient 2). ### A total of 71 women with pituitary tumors diagnosed before or during pregnancy (2 women presented with PA during pregnancy). #### patients with PA during pregnancy with ages of 32 years (patient 1), 37 years (patient 2), and 30 years (patient 3). ##### A total of 46 female patients with lactotroph PitNETs ≥ 1 cm, with at least one pregnancy after diagnosis (3 patients developed PA during pregnancy). ###### A total of 3 females diagnosed with PA in pregnancy with ages of 33 years (patient 1), 22 years (patient 2), and 29 years (patient 3). ####### A total of 5 females with PA in pregnancy with ages of 31 years (patient 1), 21 years (patient 2), 32 years (patient 3), 23 years (patient 4), and 25 years (patient 5).

**Table 2:** Introduces the data concerning additional risk factors, therapy, delivery, and maternal outcome [20,64,65,66,67,68,69,70,71,72,73,74,75,76,77,78,79,80,81,82,83,84,85,86,87,88,89,90,91,92].

Reference (Name and Number)	Additional Risk Factors	Treatment	Delivery	Maternal Outcome
Couture [64]	None	Conservative management with DA (cabergoline)	LB at 38 WG by CS	Complete recovery Resolution of pituitary adenoma
Jansssen [65]	None	Conservative treatment with DA (bromocriptine), LT4 + Hydrocortisone	LB at 40 WG by VD	Adrenal insufficiency Major decrease in size of pituitary tumor
Kita [66]	None	TSS (7 days after admission)	LB at 40 WG by CS	DI managed with 1-desamino-8-D-arginine vasopressin
Witek [67]	None	Conservative management with DA (bromocriptine) followed by TSS (at 20 WG due to visual field defects)	LB at 38 WG by CS	Complete recovery No tumor regrowth Normal pituitary function
Chegour [68]	DA (bromocriptine)	Conservative treatment with DA (cabergoline)	NA	Complete recovery: remission of symptoms and disappearance of the expansive process
Hayes [69]	DA (cabergoline) before pregnancy (discontinued when pregnancy was confirmed)	TSS	LB at term by VD	Resolution of symptoms Normal pituitary function Able to breastfeed for only 2 weeks No adenoma recurrence
Piantanida [70]	None	TSS (after delivery) 9 months after delivery cabergoline therapy was started	LB at 35 WG by urgent CS	Central hypothyroidism Total adenoma resection Hyperprolactinemia treated with cabergoline
Tandon [71]	DA (bromocriptine) before pregnancy	TSS	LB at 37 WG by CS	Transient DI postoperatively Improvement of visual symptoms
Bedford [72]	NA	NA	NA	NA
De Ycaza [73]	DA (bromocripine and cabergoline) before pregnancy (discontinued when pregnancy was confirmed)	Conservative treatment with glucocorticoid replacement and DA (cabergoline) until delivery	LB at term by VD	Tumor decreased in size after DA (cabergoline) treatment The patient had an uneventful second pregnancy
Grand’Maison [20]	Patient 1: None Patient 2: DA (cabergoline) before pregnancy (discontinued when pregnancy was confirmed)	Patient 1: Conservative management Patient 2: Conservative management and DA (cabergoline)	Patient 1: LB at 40 WG by VD Patient 2: LB at term by VD	# Patient 1: Normal pituitary function Patient 2: Normal pituitary function, diminished pituitary mass (9 × 9 mm), and uneventful second pregnancy
Watson [74]	Low-molecular-weight heparin	Conservative treatment with hydrocortisone 50 mg 6 times hourly Heparin postoperatively	LB at term by CS	Persistent hypocortisolism
Abraham [75]	None	TSS	NA	Transient postoperative DI Normal pituitary function
Annamalai [76]	DA (cabergoline) before pregnancy (discontinued when pregnancy was confirmed)	Conservative treatment with hydrocortisone and DA (cabergoline)	LB at 37 WG by CS	Resolution of PA Resolution of pituitary microadenoma Normal pituitary function
Galvão [77]	Patient 1: None Patient 2: None	Patient 1: Conservative management Patient 2: TSS during second trimester	Patient 1: LB Patient 2: LB	Patient 1: Pregnancy proceeded normally Patient 2: The patient developed DI and central hypothyroidism
Lambert [78]	NA	Both patients received conservative treatment	Patient 1: CS Patient 2: NA	Good outcome
O’Neal [79]	None	Conservative management with hydrocortisone and DA (bromocriptine) initially, followed by surgery	LB at term	DI postoperatively
Bachmeier [80]	None	TSS tumor resection PP	LB at 37 WG by CS	Resolution of symptoms Normal pituitary function postoperatively The patient was able to breastfeed
Jemel [81]	Patient 1: None Patient 2: DA (cabergoline) before pregnancy (discontinued when pregnancy was confirmed) Patient 3: None	Patient 1: Conservative management with hydrocortisone 100 mg 6 times hourly and DA in PP Patient 2: Conservative management initially, with hydrocortisone and DA, followed by TSS (3 days after admission) Patient 3: Hydrocortisone and TSS	Patient 1: LB at 37 WG Patient 2: LB at 37 WG Patient 3: LB at 38 WG	Patient 1: Regression of the pituitary mass Patient 2: NA Patient 3: Remission of symptoms
Barraud [82]	NA	Two patients underwent emergency pituitary surgery due to worsening of visual field defects The third patient underwent surgery after delivery	Patient 3: LB at 36 WG by CS	NA
Bichard [83]	NA	Conservative treatment with hydrocortisone, thyroxine, and desmopressin	LB at term by VD with forceps following induced labor	Clinically well and able to breastfeed Desmopressin and hydrocortisone requirements were reduced
Chan [84]	Acute COVID-19 infection	Initial conservative management with corticosteroids (dexamethasone) TSS: 2 days after delivery	LB at 39 WG by VD under epidural anesthesia	Central hypothyroidism and hypogonadism Possible persistence of secondary adrenal insufficiency (the patient did not undergo cortisol stimulation test)
Oguz [85]	DA (cabergoline) before pregnancy (discontinued when pregnancy was confirmed)	TSS (at 22 WG)	LB at 37 WG by CS	## Full recovery Hypothyroidism No residual tumor
Geissler [86]	gestational DM during both pregnancies	In both pregnancies: conservative steroid treatment	First presentation: LB at 36th week by CS Second presentation: LB at 34th week by CS	Complete recovery Decreased pituitary size on MRI after the first pregnancy Normal pituitary function Lack of milk production
Kanneganti [87]	None	Conservative treatment with hydrocortisone	LB at term by CS	NA
Kato [88]	Patient 1: DA treatment (terguride) before pregnancy (discontinued when pregnancy was confirmed)	Initial conservative treatment with hydrocortisone, followed by elective TSS after delivery in all three cases	Patient 1: LB at 36th week by CS Patient 2: LB at 34th week by CS Patient 3: LB at 37th week by CS	Complete recovery in all three cases
Khaldi [89]	gestational DM DA (cabergoline) before pregnancy	Conservative management with DA (bromocriptine) and hydrocortisone	LB at 28 WG by premature VD due to premature rupture of membranes Twins died on the 7th day of life	Adrenal insufficiency and central hypothyroidism Decrease in tumor size
Kuhn [90]	###	####	Patient 1: LB at term by CS Patient 2: LB by VD Patient 3: LB by VD Patient 4: LB at 38th week by CS Patient 5: LB at term by VD	Patient 1: Resolution of symptoms Patient 2: DI and hyperprolactinemia Patient 3: Resolution of headache, improvement of vision, and corticotropic deficiency Patient 4: Resolution of symptoms and able to breastfeed Patient 5: unable to breastfeed
Ye [91]	None	Conservative treatment with hydrocortisone and levothyroxine	LB at 38 + 1 WG by CS	##### Lack of lactation after delivery Regression of pituitary tumor Remission of symptoms
Sedai [92]	None	Initially conservative treatment for eclampsia (initial diagnosis) Craniotomy—tumor resection and hematoma evacuation	Maternal exitus	Exitus on the 2nd postoperative day (Initially misdiagnosed as eclampsia)

# Patient 1: The patient suffered from gestational DM and preeclampsia during previous pregnancies. Patient 2: The patient received treatment with cabergoline since 13 WG due to rapid, more than 10-fold increase in prolactin level. ## Patient was treated with cabergoline for 12 months before pregnancy. The patient suffered from an acute ischemic stroke 10 days PP. ### Patient 1: DA (cabergoline) before pregnancy (discontinued when pregnancy was confirmed). Patient 2: DA (bromocriptine) before pregnancy (discontinued when pregnancy was confirmed). Patient 3: DA (cabergoline) before and between pregnancies (discontinued when pregnancy was confirmed in all three pregnancies). Patient 4: type 1 DM, DA (cabergoline) before pregnancy (discontinued when pregnancy was confirmed). Patient 5: previous PA and DA (cabergoline) before pregnancy. #### Patient 1: TSS (second day after admission). Patient 2: Conservative treatment with hydrocortisone, LT4, desmopressin, and TSS (5 months after delivery). Patient 3: Conservative treatment with hydrocortisone and cabergoline. Patient 4: Conservative treatment with cabergoline. Patient 5: Conservative treatment with hydrocortisone and TSS (one year after delivery). ##### The patient suffered from pituitary insufficiency due to PA and developed extrapontine myelinolysis after sodium supplementation. Abbreviations: A = abortion; CS = cesarian section; DA = dopamine agonist; DI = diabetes insipidus; DM = diabetes mellitus; G = gesta; LB = live birth; NFPA = non-functioning pituitary adenoma; P = para; PA = pituitary apoplexy; PitNET = pituitary neuroendocrine tumor; PP = postpartum; TSS = transsphenoidal surgery; VD = vaginal delivery; WG = weeks of gestation.

**Table 4:** Synthesis of the most important results according to our analysis.

Parameter	Outcome
reviewed period	2012–2022
number of original studies	35
number of observational studies	7
case series	4
case reports	28
total number of patients with PA	49
PA in pregnancy/postpartum ratio	43/6
PA in pregnancy: age ranges	21–41 years
mean age at PA diagnostic in pregnancy	27.76 years
presentation during third trimester	21/43
average week of gestation	26.38
cesarean section	19/30
pre-pregnancy medication	dopamine agonists 15/43 terguride (1/43)
conservative approach	29/43
trans-sphenoidal surgery	22/43 (10/22 neurosurgery as initial approach)
number of patients with pituitary tumor not diagnosed before surgery	18/43
type of pituitary tumors	prolactinomas (26/43)
PA in postpartum: mean age at diagnosis	33 years
rate of PA in postpartum after second pregnancy	50%
timing (after delivery) of PA	5 min–12 days
rate of persistent hypopituitarism after PA in postpartum	50%

Abbreviations: PA = pituitary apoplexy.

## Data Availability

Not applicable.

## Abbreviations

A = abortion; CS = cesarian section; DA = dopamine agonist; DI = diabetes insipidus; DM = diabetes mellitus; G = gesta; GH = growth hormone; NFPA = non-functioning pituitary adenoma; N = number of patients; MRI = magnetic resonance imaging; P = para; PA = pituitary apoplexy; PitNET = pituitary neuroendocrine tumor; PP = postpartum; RCVS = reversible cerebral vasoconstrictive syndrome; TSS = trans-sphenoidal surgery; VG = vaginal delivery; WG = weeks of gestation.

## References

[1] Bi W.L., Dunn I.F., Laws E.R. (2015). Pituitary apoplexy. Endocrine.

[2] Suri H., Dougherty C. (2019). Presentation and Management of Headache in Pituitary Apoplexy. Curr. Pain Headache Rep..

[3] Barkhoudarian G., Kelly D.F. (2019). Pituitary Apoplexy. Neurosurg. Clin. N. Am..

[4] Mavridis I., Meliou M., Pyrgelis E.-S. (2017). Presenting Symptoms of Pituitary Apoplexy. J. Neurol. Surg. Part A Cent. Eur. Neurosurg..

[5] Pearce J.M. (2015). On the origins of pituitary apoplexy. Eur. Neurol..

[6] MacGregor E.A. (2012). Headache in pregnancy. Neurol. Clin..

[7] Khoo C.M., Lee K.O. (2013). Endocrine emergencies in pregnancy. Best Pract. Res. Clin. Obstet. Gynaecol..

[8] Briet C., Salenave S., Bonneville J.-F., Laws E.R., Chanson P. (2015). Pituitary Apoplexy. Endocr. Rev..

[9] Kreitschmann-Andermahr I., Siegel S., Carneiro R.W., Maubach J.M., Harbeck B., Brabant G. (2013). Headache and pituitary disease: A systematic review. Clin. Endocrinol..

[10] Hage R., Eshraghi S.R., Oyesiku N.M., Ioachimescu A.G., Newman N.J., Biousse V., Bruce B.B. (2016). Third, Fourth, and Sixth Cranial Nerve Palsies in Pituitary Apoplexy. World Neurosurg..

[11] Ishii M. (2017). Endocrine Emergencies With Neurologic Manifestations. Contin. Lifelong Learn. Neurol..

[12] Takeda R., Demura M., Sugimura Y., Miyamori I., Konoshita T., Yamamoto H. (2021). Pregnancy-associated diabetes insipidus in Japan—A review based on quoting from the literatures reported during the period from 1982 to 2019. Endocr. J..

[13] Johnston P.C., Hamrahian A.H., Weil R.J., Kennedy L. (2015). Pituitary tumor apoplexy. J. Clin. Neurosci..

[14] Donegan D., Erickson D. (2022). Revisiting Pituitary Apoplexy. J. Endocr. Soc..

[15] Araujo-Castro M., Berrocal V.R., Pascual-Corrales E. (2020). Pituitary tumors: Epidemiology and clinical presentation spectrum. Hormones.

[16] Vargas G., Gonzalez B., Guinto G., Mendoza V., López-Félix B., Zepeda E., Mercado M. (2014). Pituitary apoplexy in nonfunctioning pituitary macroadenomas: A case-control study. Endocr. Pract..

[17] Sarwar K.N., Huda M.S.B., Van De Velde V., Hopkins L., Luck S., Preston R., McGowan B., Carroll P.V., Powrie J.K. (2013). The prevalence and natural history of pituitary hemorrhage in prolactinoma. J. Clin. Endocrinol. Metab..

[18] Nawar R.N., AbdelMannan D., Selman W.R., Arafah B.M. (2008). Pituitary tumor apoplexy: A review. J. Intensiv. Care Med..

[19] Muthukumar N. (2020). Pituitary Apoplexy: A Comprehensive Review. Neurol. India.

[20] Grand’Maison S., Weber F., Bédard M.-J., Mahone M., Godbout A. (2015). Pituitary apoplexy in pregnancy: A case series and literature review. Obstet. Med..

[21] Semple P.L., Jane J.A., Laws E.R. (2007). Clinical relevance of precipitating factors in pituitary apoplexy. Neurosurgery.

[22] Wildemberg L.E., Glezer A., Bronstein M.D., Gadelha M.R. (2018). Apoplexy in nonfunctioning pituitary adenomas. Pituitary.

[23] Wichlińska-Lubińska M., Kozera G. (2019). Pituitary apoplexy. Neurol. I Neurochir. Pol..

[24] Pivonello R., De Martino M.C., Auriemma R.S., Alviggi C., Grasso L.F.S., Cozzolino A., De Leo M., DE Placido G., Colao A., Lombardi G. (2014). Pituitary tumors and pregnancy: The interplay between a pathologic condition and a physiologic status. J. Endocrinol. Investig..

[25] Scheithauer B.W., Sano T., Kovacs K.T., Young W.F., Ryan N., Randall R.V. (1990). The pituitary gland in pregnancy: A clinicopathologic and immunohistochemical study of 69 cases. Mayo Clin. Proc..

[26] Huang W., Molitch M.E. (2019). Pituitary Tumors in Pregnancy. Endocrinol. Metab. Clin. N. Am..

[27] Cardoso E.R., Peterson E.W. (1984). Pituitary apoplexy: A review. Neurosurgery.

[28] Qaiser R., Black P. (2007). Neurosurgery in pregnancy. Semin. Neurol..

[29] Molitch M.E. (2003). Pituitary tumors and pregnancy. Growth Horm. IGF Res..

[30] Frise C.J., Williamson C. (2013). Endocrine disease in pregnancy. Clin. Med. J. R. Coll. Physicians Lond..

[31] Araujo P.B., Neto L.V., Gadelha M.R. (2015). Pituitary Tumor Management in Pregnancy. Endocrinol. Metab. Clin. N. Am..

[32] Woodmansee W.W. (2019). Pituitary Disorders in Pregnancy. Neurol. Clin..

[33] Laway B.A., Mir S.A. (2013). Pregnancy and pituitary disorders: Challenges in diagnosis and management. Indian J. Endocrinol. Metab..

[34] Valassi E. (2021). Pituitary disease and pregnancy. Endocrinol. Diabetes Nutr..

[35] Alex A., Bhandary E., McGuire K.P. (2020). Anatomy and physiology of the breast during pregnancy and lactation. Adv. Exp. Med. Biol..

[36] Chrisoulidou A., Boudina M., Karavitaki N., Bili E.W.J. (2015). Pituitary disorders in pregnancy. Hormones.

[37] Grattan D.R. (2015). 60 years of neuroendocrinology: The hypothalamo-prolactin axis. J. Endocrinol..

[38] Honegger J., Nasi-Kordhishti I., Aboutaha N., Giese S. (2020). Surgery for prolactinomas: A better choice?. Pituitary.

[39] Biagetti B., Simò R. (2022). Pituitary Apoplexy: Risk Factors and Underlying Molecular Mechanisms. Int. J. Mol. Sci..

[40] Toossi S., Moheet A.M. (2019). Intracerebral Hemorrhage in Women: A Review with Special Attention to Pregnancy and the Post-Partum Period. Neurocritical Care.

[41] Iuliano S., Laws E.R. (2011). Management of pituitary tumors in pregnancy. Semin. Neurol..

[42] Petersenn S. (2018). Pituitary disease management during pregnancy: An overview. Minerva Endocrinol..

[43] Schoen J., Campbell R.L., Sadosty A.T. (2015). Headache in pregnancy: An approach to emergency department evaluation and management. West J. Emerg. Med..

[44] Guryildirim M., Kontzialis M., Ozen M., Kocak M. (2019). Acute Headache in the Emergency Setting. RadioGraphics.

[45] Negro A., Delaruelle Z., Ivanova T.A., Khan S., Ornello R., Raffaelli B., Terrin A., Reuter U., Mitsikostas D.D., European Headache Federation School of Advanced Studies (EHF-SAS) (2017). Headache and pregnancy: A systematic review. J. Headache Pain.

[46] Burch R. (2019). Headache in Pregnancy and the Puerperium. Neurol. Clin..

[47] Bamfo J.E.A.K., Sharif S., Donnelly T., Cohen M.A., Golara M. (2011). A case of pituitary apoplexy masquerading as hyperemesis gravidarum. J. Obstet. Gynaecol..

[48] Motivala S., Gologorsky Y., Kostandinov J., Post K.D. (2011). Pituitary disorders during pregnancy. Endocrinol. Metab. Clin. N. Am..

[49] Inder W.J., Jang C. (2022). Treatment of Prolactinoma. Medicina.

[50] Zak I.T., Dulai H.S., Kish K.K. (2007). Imaging of neurologic disorders associated with pregnancy and the postpartum period. Radiographics.

[51] Zamora C., Castillo M. (2022). Role of MRI and CT in the Evaluation of Headache in Pregnancy and the Postpartum Period. Neurol. Clin..

[52] E Baldeweg S., Vanderpump M., Drake W., Reddy N., Markey A., Plant G.T., Powell M., Sinha S., Wass J., Society for Endocrinology Clinical Committee (2016). Society for endocrinology endocrine emergency guidance: Emergency management of pituitary apoplexy in adult patients. Endocr. Connect..

[53] Rajasekaran S., Vanderpump M., Baldeweg S., Drake W., Reddy N., Lanyon M., Markey A., Plant G., Powell M., Sinha S. (2011). UK guidelines for the management of pituitary apoplexy Pituitary Apoplexy Guidelines Development Group: May 2010. Clin. Endocrinol..

[54] Chanson P., Raverot G., Castinetti F., Cortet-Rudelli C., Galland F., Salenave S., French Endocrinology Society Non-Functioning Pituitary Adenoma Work-Group (2015). Management of clinically non-functioning pituitary adenoma. Ann. Endocrinol..

[55] Glezer A., Bronstein M.D. (2018). Prolactinomas: How to handle prior to and during pregnancy?. Minerva Endocrinol..

[56] Glezer A., Bronstein M.D. (2020). Prolactinomas in pregnancy: Considerations before conception and during pregnancy. Pituitary.

[57] Luger A., A Broersen L.H., Biermasz N.R., Biller B.M.K., Buchfelder M., Chanson P., Jorgensen J.O.L., Kelestimur F., Llahana S., Maiter D. (2021). ESE Clinical Practice Guideline on functioning and nonfunctioning pituitary adenomas in pregnancy. Eur. J. Endocrinol..

[58] E Molitch M. (2015). Endocrinology in pregnancy: Management of the pregnant patient with a prolactinoma. Eur. J. Endocrinol..

[59] Graillon T., Cuny T., Castinetti F., Courbière B., Cousin M., Albarel F., Morange I., Bruder N., Brue T., Dufour H. (2020). Surgical indications for pituitary tumors during pregnancy: A literature review. Pituitary.

[60] Karaca Z., Tanriverdi F., Unluhizarci K., Kelestimur F. (2010). Pregnancy and pituitary disorders. Eur. J. Endocrinol..

[61] Lamba N., Noormohamed N., Simjian T., Alsheikh M.Y., Jamal A., Doucette J., Zaidi H., Smith T.R., Mekary R.A. (2019). Fertility after transsphenoidal surgery in patients with prolactinomas: A meta-analysis. Clin. Neurol. Neurosurg..

[62] Chowdhury T., Chowdhury M., Schaller B., Cappellani R.B., Daya J. (2013). Perioperative considerations for neurosurgical procedures in the gravid patient: Continuing Professional Development. Can. J. Anaesth..

[63] Xia Y., Ma X., Griffiths B.B., Luo Y. (2018). Neurosurgical anesthesia for a pregnant woman with macroprolactinoma: A case report. Medicine.

[64] Couture N., Aris-Jilwan N., Serri O. (2012). Apoplexy of a microprolactinoma during pregnancy: Case report and review of literature. Endocr. Pract..

[65] Janssen N.M., Dreyer K., Van Der Weiden R.M.F. (2012). Management of pituitary tumour apoplexy with bromocriptine in pregnancy. JRSM Short Rep..

[66] Kita D., Hayashi Y., Sano H., Takamura T., Hayashi Y., Tachibana O., Hamada J.-I. (2012). Postoperative diabetes insipidus associated with pituitary apoplexy during pregnancy. Neuro Endocrinol. Lett..

[67] Witek P., Zieliński G., Maksymowicz M., Zgliczyński W. (2012). Transsphenoidal surgery for a life-threatening prolactinoma apoplexy during pregnancy. Neuro Endocrinol. Lett..

[68] Chegour H., El Ansari N. (2014). Pituitary apoplexy during pregnancy. Pan Afr. Med. J..

[69] Hayes A.R., O’Sullivan A.J., A Davies M. (2014). A case of pituitary apoplexy in pregnancy. Endocrinol. Diabetes Metab. Case Rep..

[70] Piantanida E., Gallo D., Lombardi V., Tanda M.L., Lai A., Ghezzi F., Minotto R., Tabano A., Cerati M., Azzolini C. (2014). Pituitary apoplexy during pregnancy: A rare, but dangerous headache. J. Endocrinol. Investig..

[71] Tandon A., Alzate J., LaSala P., Fried M.P. (2014). Endoscopic Endonasal Transsphenoidal Resection for Pituitary Apoplexy during the Third Trimester of Pregnancy. Surg. Res. Pract..

[72] Bedford J., Dassan P., Harvie M., Mehta S. (2015). An unusual cause of headache in pregnancy. BMJ.

[73] De Ycaza A.E., Chang A.Y., Jensen J.R., Khan Z., Erickson D. (2015). Approach to the management of rare clinical presentations of macroprolactinomas in reproductive-aged women. Case Rep. Women’s Health.

[74] Watson V. (2015). An unexpected headache: Pituitary apoplexy in a pregnant woman on anticoagulation. BMJ Case Rep..

[75] Abraham R.R., E Pollitzer R., Gokden M., A Goulden P. (2016). Spontaneous pituitary apoplexy during the second trimester of pregnancy, with sensory loss. BMJ Case Rep..

[76] Annamalai A.K., Jeyachitra G., Jeyamithra A., Ganeshkumar M., Srinivasan K.G., Gurnell M. (2017). Gestational Pituitary Apoplexy Prevalence of Islet Autoantibodies in Type 1 Diabetes. Indian J. Endocrinol. Metab..

[77] Galvão A., Gonçalves D., Moreira M., Inocêncio G., Silva C., Braga J. (2017). Prolactinoma and pregnancy—A series of cases including pituitary apoplexy. J. Obstet. Gynaecol..

[78] Lambert K., Rees K., Seed P.T., Dhanjal M.K., Knight M., McCance D.R., Williamson C. (2017). Macroprolactinomas and Nonfunctioning Pituitary Adenomas and Pregnancy Outcomes. Obstet. Gynecol..

[79] O’Neal M.A. (2017). Headaches complicating pregnancy and the postpartum period. Pract. Neurol..

[80] Bachmeier C.A.E., Snell C., Morton A. (2019). Visual loss in pregnancy. BMJ Case Rep..

[81] Jemel M., Kandara H., Riahi M., Gharbi R., Nagi S., Kamoun I. (2019). Gestational pituitary apoplexy: Case series and review of the literature. J. Gynecol. Obstet. Hum. Reprod..

[82] Barraud S., Guédra L., Delemer B., Raverot G., Ancelle D., Fèvre A., Jouanneau E., Litré C., Wolak-Thierry A., Borson-Chazot F. (2020). Evolution of macroprolactinomas during pregnancy: A cohort study of 85 pregnancies. Clin. Endocrinol..

[83] Bichard L.K., Torpy D.J. (2020). Diabetes insipidus complicating apoplexy during pregnancy: The potential use of copeptin. Intern. Med. J..

[84] Chan J., Gregory K.D., Smithson S.S., Naqvi M., Mamelak A.N. (2020). Pituitary apoplexy associated with acute COVID-19 infection and pregnancy. Pituitary.

[85] Oguz S.H., Soylemezoglu F., Dagdelen S., Erbas T. (2020). A case of atypical macroprolactinoma presenting with pituitary apoplexy during pregnancy and review of the literature. Gynecol. Endocrinol..

[86] Geissler F., Hoesli I., Bernasconi M.T. (2021). Recurrent pituitary apoplexy in pregnancy. BMJ Case Rep..

[87] Kanneganti A., Lwin S., Su L.L. (2021). Pituitary Apoplexy in Pregnancy. J. Obstet. Gynaecol. Can..

[88] Kato Y., Ogawa Y., Tominaga T. (2021). Treatment and therapeutic strategies for pituitary apoplexy in pregnancy: A case series. J. Med. Case Rep..

[89] Khaldi S., Saad G., Elfekih H., Ben Abdelkrim A., Ach T., Kacem M., Chaieb M., Maaroufi A., Hasni Y., Ach K. (2021). Pituitary apoplexy of a giant prolactinoma during pregnancy. Gynecol. Endocrinol..

[90] Kuhn E., A Weinreich A., Biermasz N.R., Jorgensen J.O.L., Chanson P. (2021). Apoplexy of microprolactinomas during pregnancy: Report of five cases and review of the literature. Eur. J. Endocrinol..

[91] Ye W., Huang W., Chen L., Yao C., Sheng S., Liu Z., Xue C., Xing W. (2021). Pituitary tumor apoplexy associated with extrapontine myelinolysis during pregnancy: A case report. Medicine.

[92] Sedai H., Shrestha S., Poddar E., Sharma P., Dahal D., Khatiwada P., Pradhanang A. (2022). Delayed identification of massive pituitary apoplexy in pregnancy: A case report. Int. J. Surg. Case Rep..

[93] Mathur D., Lim L.F.M., Mathur M., Sng B.L. (2014). pituitary apoplexy with reversible cerebral vasoconstrictive syndrome after spinal anaesthesia for emergency caesarean section: An uncommon cause for postpartum headache. Anaesth. Intensiv. Care.

[94] Raina S., Jearth V., Sharma A., Sharma R., Mistry K. (2015). Postpartum pituitary apoplexy with isolated oculomotor nerve palsy: A rare medical emergency. J. Neurosci. Rural. Pract..

[95] Dias R., Ferreira C., Mendes B., Marvão J., Lages N.M.H. (2021). Postpartum headache after epidural anaesthesia: Who to blame?. Rev. Esp. Anestesiol. Reanim..

[96] Hoang V.T., Hoang T.H., Nguyen T.T.T., Chansomphou V., Hoang D.T. (2022). Pituitary Apoplexy and Subdural Hematoma after Caesarean Section. Case Rep. Obstet. Gynecol..

[97] Pop L.G., Ilian A., Georgescu T., Bacalbasa N., Balescu I., Toader O.D. (2022). Pituitary adenoma apoplexy in pregnancy: Case report and literature review. Exp. Ther. Med..

[98] Ducros A., Bousser M.-G. (2009). Reversible cerebral vasoconstriction syndrome. Pract. Neurol..

[99] Ananthakrishnan S. (2020). Gestational diabetes insipidus: Diagnosis and management. Best Pract. Res. Clin. Endocrinol. Metab..

[100] Trandafir A.I., Petrova E., Ghemigian A., Valea A., Carsote M., Sandru F. (2021). Emergency hypophysectomy for pituitary apoplexy in a previously undiagnosed case of acromegaly. Rom. J. Emerg. Surg..

[101] Carsote M., Valea A., Dumitrascu M.C., Albu S.E., Sandru F. (2019). Pituitary non-functioning macroadenomas: If and when to recommend surgery. Rom. Med. J..

[102] Arnold M.A., Barbero J.M.R., Pradilla G., Wise S.K. (2022). Pituitary Gland Surgical Emergencies: The Role of Endoscopic Intervention. Otolaryngol. Clin. N. Am..

[103] (2018). Diabetes insipidus in pregnancy: How to advice the patient?. Minerva Endocrinol..

[104] Tomkins M., Lawless S., Martin-Grace J., Sherlock M., Thompson C.J. (2022). Diagnosis and Management of Central Diabetes Insipidus in Adults. J. Clin. Endocrinol. Metab..

[105] Mutter C.M., Smith T., Menze O., Zakharia M., Nguyen H. (2021). Diabetes Insipidus: Pathogenesis, Diagnosis, and Clinical Management. Cureus.

[106] Ribas M.Z., Paticcié G.F., de Medeiros S.D.P., Veras A.D.O., Noleto F.M., dos Santos J.C.C. (2023). Reversible cerebral vasoconstriction syndrome: Literature review. Egypt. J. Neurol. Psychiatry Neurosurg..

[107] Chen S.-P., Wang S.-J. (2022). Pathophysiology of reversible cerebral vasoconstriction syndrome. J. Biomed. Sci..

[108] Perillo T., Paolella C., Perrotta G., Serino A., Caranci F., Manto A. (2022). Reversible cerebral vasoconstriction syndrome: Review of neuroimaging findings. La Radiol. Med..

[109] Daskalakis G., Pergialiotis V., Domellöf M., Ehrhardt H., Di Renzo G.C., Koç E., Malamitsi-Puchner A., Kacerovsky M., Modi N., Shennan A. (2023). European guidelines on perinatal care: Corticosteroids for women at risk of preterm birth. J. Matern. Neonatal Med..

[110] Dumitru N., Panait C.G., Cocolos A., Sandru F., Valea A., Carsote M., Ghemigian A. (2023). Autoimmune thyroid disease and pregnancy, what could be a common factor?. Rom. J. Clin. Res..

[111] Drui D., Briet C., Guerin C., Lugat A., Borson-Chazot F., Grunenwald S. (2022). SFE-AFCE-SFMN 2022 Consensus on the management of thyroid nodules: Thyroid nodules and pregnancy. Ann. Endocrinol..

[112] Honegger J., Giese S., Nasi-Kordhishti I., Donegan D.M. (2023). Pregnancy-related hypophysitis revisited. Eur. J. Endocrinol..

[113] Laway B.A. (2023). Sheehan syndrome: Cardiovascular and metabolic comorbidities. Front. Endocrinol..

[114] Zain A., Sivakumar A., Akah O., Shiza S.T., Mahadevaiah A., Khan A. (2022). A Rare Case of Sheehan Syndrome with Cardiac Tamponade. Cureus.

[115] Cherian K.E., Kapoor N., Paul T.V., Asha H.S. (2021). Functioning Endocrine Tumors in Pregnancy: Diagnostic and Therapeutic Challenges. Indian J. Endocrinol. Metab..

[116] Nistor C.-E., Pantile D., Stanciu-Gavan C., Ciuche A., Moldovan H. (2022). Diagnostic and Therapeutic Characteristics in Patients with Pneumotorax Associated with COVID-19 versus Non-COVID-19 Pneumotorax. Medicina.

[117] Mirza S.A., Sheikh A.A.E., Barbera M., Ijaz Z., Javaid M.A., Shekhar R., Pal S., Sheikh A.B. (2022). COVID-19 and the Endocrine System: A Review of the Current Information and Misinformation. Infect. Dis. Rep..

[118] Nistor C.E., Gavan C.S., Pantile D., Tanase N.V., Ciuche A. (2022). Cervico-Thoracic Air Collections in COVID-19 Pneumonia Patients—Our Experience and Brief Review. Chirurgia.

[119] Garg M.K., Gopalakrishnan M., Yadav P., Misra S. (2020). Endocrine Involvement in COVID-19: Mechanisms, Clinical Features, and Implications for Care. Indian J. Endocrinol. Metab..

[120] Zhu G., Liu L., Huang X., Li D., Zhu Y., Lu X., Du M. (2022). The risk of intrauterine exposure to SARS-CoV-2 in female COVID-19 patients: A comprehensive review. Am. J. Reprod. Immunol..

